# Is carotid artery screening obligatory in young patients undergoing coronary artery bypass grafting?

**DOI:** 10.1186/1749-8090-8-S1-O174

**Published:** 2013-09-11

**Authors:** S Chlapoutakis, S Siminelakis, S Sismanidis, I Mpeis, E Apostolakis

**Affiliations:** 1 Cardiothoracic Surgery Department, Ioannina, Greece

## Background

Postoperative complications from the Central Nervous System after Coronary Artery Bypass Grafting Operations (CABG) can range from transient neurologic or psychological changes to massive brain infarction and irreversible coma. The risk of stoke after CABG is approximately 2%. Multiple mechanisms such as brain hypoperfusion during or after the operation, carotid stenosis and embolization can lead to these complications.

## Methods

635 patients with risk factors for Cerebral Vascular Accident (CVA) such as history of stroke, peripheral vascular disease (PVD) and age over 65 years old compared to 331 patients <65 years old without any of these risk factors. All patients underwent CABG procedure and carotid endarterectomy at the same time when was necessary. Other cardiac operations excluded. The following risk factors for CVA were considered: age>65 years old, history of CVA, history of PVD. All patients had a preoperative carotid triplex. For practical reasons the Carotid Artery Stenosis was defund as significant> 50-75% and severe > 75%. EuroSCORE I calculated as well for all patients in order to calculate the postoperative mortality score.

## Results

From the low risk group (311 patients), 33 (10%) were found to have significant carotid stenosis and 5 had severe stenosis and underwent Carotid endarterectomy and CABG at the same time. None of them suffered from postoperative stroke and one patient died due to chest infection. From the high-risk group (635 patients) 15 (2,36%) developed transient neurologic symptoms and 6 (0,94%) developed severe postoperative stroke and 2 (0,31%) died.

## Conclusion

Because of the catastrophic neurological complications in patients undergo Coronary Artery Bypass operations even if they are low risk patients for CVA we suggest to have a preoperative carotid triplex in order to find asymptomatic lesions and take all the perioperative precautions. That will improve the outcome and will reduce more the morbidity and mortality.

